# Time-varying effect of drunk driving regulations on road traffic mortality in Guangzhou, China: an interrupted time-series analysis

**DOI:** 10.1186/s12889-021-11958-4

**Published:** 2021-10-19

**Authors:** Xiao-Han Xu, Hang Dong, Li Li, Zhou Yang, Guo-Zhen Lin, Chun-Quan Ou

**Affiliations:** 1 State Key Laboratory of Organ Failure Research, Department of Biostatistics, Guangdong Provincial Key Laboratory of Tropical Disease Research, School of Public Health, Southern Medical University, Guangzhou, 510515 China; 2 Guangzhou Center for Disease Control and Prevention, Guangzhou, 510440 Guangdong China

**Keywords:** Road traffic deaths, Drunk driving regulations, China, Interrupted time-series analysis, Quasi-Poisson regression model

## Abstract

**Background:**

China has introduced a series of stricter policies to criminalize drunk driving and increase penalties since May 2011. However, there is no previous study examining the time-varying impacts of drunk driving regulations on road traffic fatalities based on daily data.

**Methods:**

We collected 6536 individual data of road traffic deaths (RTDs) in Guangzhou from 2008 to 2018. The quasi-Poisson regression models with an inclusion of the intervention variable and the interaction of intervention variable and a function of time were used to quantify the time-varying effects of these regulations.

**Results:**

During the 11-year study period, the number of population and motor vehicles showed a steady upward trend. However, the population- and motor vehicles- standardized RTDs rose steadily before May 2011, the criminalizing drunk driving intervention was implemented and gradually declined after that. The new drunk driving intervention were associated with an average risk reduction of RTDs (ER = -9.01, 95% eCI: − 10.05% to − 7.62%) during the 7.7 years after May 2011. On average, 75.82 (95% eCI, 54.06 to 92.04) RTDs per 1 million population annually were prevented due to the drunk driving intervention.

**Conclusion:**

These findings would provide important implications for the development of integrated intervention measures in China and other countries attempting to reduce traffic fatalities by stricter regulations on drunk driving.

**Supplementary Information:**

The online version contains supplementary material available at 10.1186/s12889-021-11958-4.

## Background

Road safety is a major public health issue [1]. Approximately 1.35 million people are killed in road traffic crashes each year [2]. The road traffic crashes are the eighth leading cause of death for the entire population and top one cause of death among children and young adults under 30 years [1]. Drunk driving is a major risk factor of road traffic crashes globally [3, 4]. It is estimated that nearly 5–35% of all road traffic deaths (RTDs) are alcohol-related [1]. Compared with high-income countries, the incidence of drunk driving is even higher in low- and middle-income countries, with approximately 33–69% of fatally injured drivers drinking before their crash [5].

Previous studies have shown that regulations related to drunk driving reduced the number of road traffic injuries and deaths in some countries, such as Canada, Chile, and United States and China [6–9]. These previous studies commonly used the autoregressive integrated moving average with exogenous variable (i.e. ARIMAX) model [6, 7, 10] or generalized additive model [11–13] to evaluate the impact of drunk driving laws. The generalized additive model is a more flexible alternative to ARIMAX. For instance, various distributions that are appropriate for counts data can be chosen, such as quasi-Poisson distribution and negative binomial distribution [11, 14]. In addition, existing studies often examined the time-invariant intervention effect based on weekly/monthly/annual instead of daily road traffic-related data [7, 8, 15]. However, the effect of drunk driving regulations is very likely to change over time as relevant regulations may be introduced successively and the strength of regulation enforcement might vary across time. To our knowledge, there is no previous study examining the time-varying impacts of drunk driving regulations on road traffic fatalities based on daily data for a long period. Estimating the intervention effect using the mortality data for a whole population rather than surveillance points may reduce selection bias [16]. Moreover, the impact of drunk driving regulation on mortality may differ in subpopulations by gender and age group [12], while the disparities by educational attainment, occupational group, and type of road user are lack of investigation. Previous studies commonly used excess risk (ER) to reflect the impact of drunk driving regulation [11, 12], while the indicator only reflects the relative risk of RTD after the intervention compared with pre-intervention period. Excess road traffic mortality rate (EMR) of RTD attributable to drunk driving regulation can present the absolute impact of the intervention.

As the largest developing country, China is experiencing rapid urbanization and motorization. Mainland China recorded approximately 262,000 RTDs in population of about 1.4 billion in 2017, accounting for approximate 21% of global RTDs [17]. That is, the road traffic mortality in China was slightly higher than the global average level (17.8 vs 16.7 per 100,000 population). Therefore, China’s progress in reducing RTDs would have a substantial impact on achieving global road traffic safety targets. To effectively reduce the adverse consequences of drunk driving, the Chinese government has adopted increasingly strict regulations and the regulation enforcement has been enhanced significantly. The landmark amendment of criminalizing driving after drunkenness (blood alcohol concentration (BAC) ≥ 0.08%) and increasing penalties for drunk driving (0.02% ≤ BAC < 0.08%) beginning on May 1, 2011, was one of the most important interventions to combat drunk driving in China [8, 18]. Evaluation of the effectiveness of drunk driving regulations is of great significance for policy making and adjustment in China and other countries.

We conducted an interrupted time-series (ITS) study in Guangzhou. Guangzhou is one of the fastest-growing economies in China, with a high population density of 1248/km^2^ in 2018 and the total number of civil motor vehicles has surged up from 1.2 million in 2008 to 2.6 million in 2018. Based on daily data of RTDs for the whole population from 2008 to 2018 in Guangzhou, China, this study aimed to quantitatively evaluate the effects of the new drunk driving regulations implemented since May 2011 on road traffic fatalities. To better present the public health significance of the regulations, we used excess risk and excess road traffic mortality rate of RTDs. The main aim was to estimate the intervention effect for the whole population with secondary aims being the explorations of subpopulation stratified by individual characteristics (i.e., sex, age group, educational attainment, occupational group, and type of road user).

## Methods

### Data collection

Guangzhou has a unified and unique death registration system that records all of the deaths from the whole population. And the death data were regularly cross-checked with the vital registration system operated by Guangzhou Municipality Public Security Bureau to update some delayed or unreported death registration. From Guangzhou Center for Disease Control and Prevention, we collected individual death data for all permanent residents of Guangzhou during 2008–2018, including cause of death, sex, date of birth, date of death, educational attainment, and occupational group. The annual numbers of permanent residents and motor vehicles were obtained from the statistical yearbook of the Guangzhou statistics bureau. The surface meteorological information of daily temperature was derived from the China Meteorological Administration (http://data.cma.cn/). We used the International Statistical Classification of Diseases and Related Health Problems 10th Revision to classify the road traffic death and considered five types of road user: pedestrian (V01-V04, V06-V09), pedal cyclist (V10-V19), motorcyclist (V20-V29), occupant (V30-V79, V87) and other or unspecified road user (V80, V82, V89). All death records were aggregated into daily counts of RTDs and stratified by sex, age group (< 16 years, 16–64 years, and ≥ 65 years), educational attainment (primary school or below, and secondary school or above), occupational group (unemployed, blue-collar workers, and white-collar workers), and type of road user. The study proposal was approved by the Ethics Committee of Guangzhou Center for Disease Control and Prevention.

### Statistical methods

We standardized RTDs, accounting for the changes in population size and number of motor vehicles, to obtain the RTDs per 1 million population and 1 million motor vehicles (sRTD represents standardized RTDs in the remaining of the paper). The mainbody of the new drunk driving intervention were implemented in May 2011 and other subsequent detailed measures have been taken after 2011 to enforce the implement of the intervention. Here, we examined the effects of a series intervention measures which were implemented since May 2011. We used the ITS design to explore the association between sRTD and the drunk driving regulations. We modeled daily RTDs using quasi-Poisson regression models, with an adjustment of long-term and seasonal trends and a covariate of ambient temperature. The model (I) formula was specified as follows:

$$ {\displaystyle \begin{array}{c}\mathrm{Log}\left(\mathrm{E}\left[{Y}_t\right]\right)=\mathrm{offset}\left(\mathrm{Log}(Pop)+\mathrm{Log}(Car)\right)+{\upbeta}_0\\ {}+\sum \limits_{\theta =1}^k\left[{\beta}_{1\theta}\sin\;\left(\frac{2\theta \pi t}{T}\right)+{\beta}_{2\theta}\cos\;\left(\frac{2\theta \pi t}{T}\right)\right]+ ns\;\left({temp}_t,3\right)+{\beta}_3\;{Dow}_t\\ {}+{\beta}_4{Holiday}_t+{\beta}_5{X}_t+{\beta}_6t+{\beta}_7{X}_tt\end{array}} $$

where *Y*_*t*_ is the number of RTDs in day *t* (*t* = 1, 2, …, 4018); the regression coefficients of the logarithm of population and motor vehicles were forced to be 1, so that the fitted model was used to explore the influential factors of sRTD; the paired sine and cosine functions were used to fit the seasonality of sRTD; *T* is 365.25 and *k* was chosen by the mass spectrogram (*k* = 1); *ns(temp*_*t*_*)* denotes the natural cubic spline of daily temperature with three degrees of freedom (*df*s) and the *df*s were chosen by minimizing the value of Akaike’s Information Criterion for the quasi-Poisson regression model. *Dow*_*t*_ is the indicator of the day of the week and *Holiday*_*t*_ is a categorical variable of public holiday (i.e., 0 for non-holiday, 1 for Spring Festival, and 2 for other holidays), *X*_*t*_ takes values of 0 and 1 indicating the period before and after May 1, 2011, respectively, *X*_*t*_*t* denotes the interaction term of *X*_*t*_ and time to consider the time-varying effects of drunk driving regulations.

To better understand the public health significance of the new drunk driving intervention, we estimated ER and EMR to reflect the impacts of the intervention. The ER of sRTD at time *t* was estimated as: ${\mathrm{ER}}_t=\left(\exp\;\left({\hat{\upbeta}}_5+{\hat{\upbeta}}_7\times t\right)\hbox{-} 1\right)\times 100\%$, where *t* represents the value of time in the Model (I). In addition, we provided the estimates of ERs at three time points: November 19, 2013, June 10, 2016, and December 31, 2018 which were the ending dates of the three equal-time intervals of the post-intervention period. ER represents the percentage change in the relative risk of sRTD associated with the drunk driving intervention. The EMR was estimated as: $\left({\sum}_{t=1217}^{t=4018}\left({D}_t-D{0}_t\right)/ Pop\right)\times 1,000,000/N$, where *D*_*t*_ is the predicted number of RTDs under the factual scenario in post-intervention time *t*; *D0*_*t*_ is the predicted number of RTDs under the counterfactual scenario that the intervention was not implemented, and *N* is the number of post-intervention years under study. EMR represents the average annual number of RTDs that were changed per 1 million individuals due to the drunk driving intervention. We used Monte Carlo simulation to estimate the 95% empirical confidence intervals (eCIs) of regression coefficients which were assumed to follow a multivariate normal distribution. And then ER and EMR were estimated accordingly.

Furthermore, we conducted the subgroup analyses by sex, age group, educational attainment, occupational group, and type of road user. Plots of partial autocorrelation functions of residuals of the fitted models are presented for checking the adequateness of the model fitting.

We conducted two sensitivity analyses to evaluate the robustness of the main results. First, we fitted the model using the monthly number of RTDs, in which day *t* in the Model I was replaced with month *t* (*t* = 1, 2, …, 132), and the *Dow*_*t*_ and *Holiday*_*t*_ variables were removed. Second, we explored the potential nonlinear pattern of intervention effect over time, the interaction term of intervention variable *X*_*t*_ and a natural cubic spline function of time were added into the Model (I) to obtain the following Model (II):

$$ {\displaystyle \begin{array}{c}\mathrm{Log}\left(\mathrm{E}\left[{Y}_t\right]\right)=\mathrm{offset}\left(\mathrm{Log}(Pop)+\mathrm{Log}(Car)\right)+{\upbeta}_0\\ {}+\sum \limits_{\theta =1}^k\left[{\beta}_{1\theta}\sin\;\left(\frac{2\theta \pi t}{T}\right)+{\beta}_{2\theta}\cos\;\left(\frac{2\theta \pi t}{T}\right)\right]+ ns\;\left({temp}_t,3\right)+{\beta}_3\;{Dow}_t\\ {}+{\beta}_4{Holiday}_t+{\beta}_5{X}_t+{\beta}_6t+{\beta}_7{X}_t\times ns\left(t,6\right)\end{array}} $$

Where *X*_*t*_ × *ns(t,6)* denotes the interaction term of intervention variable and the natural cubic spline function of time. Six is the *df*s for the natural cubic spline.

All statistical analyses were completed in R 4.0.3 (R Foundation for Statistical Computing). The main package used was “mgcv”. Statistical significance was set at *P* < 0.05 (two-sided). The R code for the main model was available in Additional file 1.

## Results

**Fig. 1:**
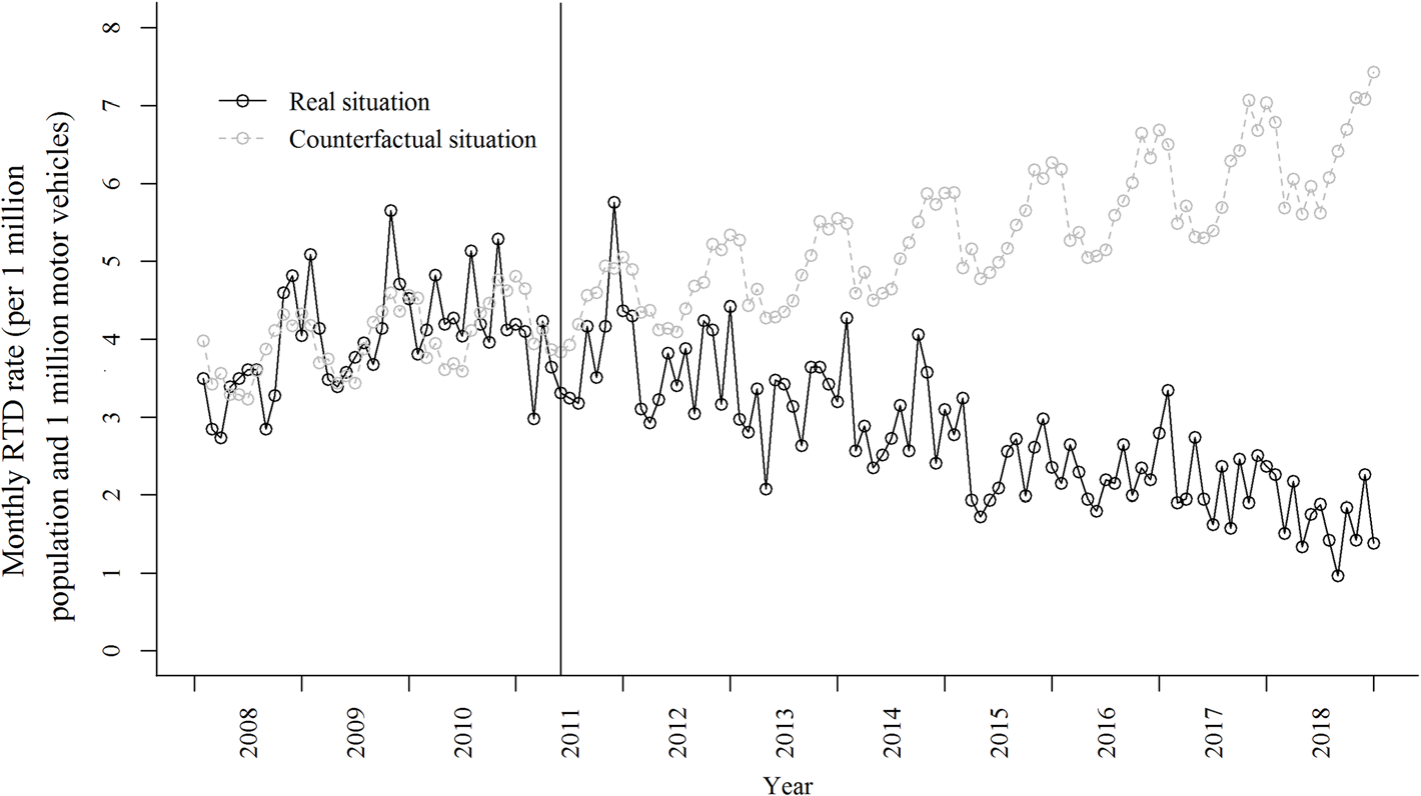
Standardized monthly road traffic deaths in Guangzhou, China, 2008–2018. RTDs: road traffic deaths. The solid vertical line shows the timing of draft amendment to Criminal Law came into force in May 2011

Table 1 presents the basic descriptive information of RTDs in Guangzhou, China. A total of 6536 RTDs were recorded from January 1, 2008 through December 31, 2018. During the 11-year study period, the number of population and motor vehicles showed a steady upward trend. However, the sRTD changed over time, rising steadily in the pre-intervention period and gradually decreasing after May 2011 (Fig. 1). The annuals RTDs increased from 42.8 in 2008 to 52.2 per 1 million population and 1 million vehicles in 2010 and then decreased markedly to 20.2 per 1 million population and 1 million vehicles in 2018.

**Table 1:** Descriptive statistics of road traffic deaths, population and motor vehicles in Guangzhou, China, 2008–2018

Year	Annual number of RTDs	Registered population (× 1 million)	Number of motor vehicles (× 1 million)	Annual rate of RTDs (per 1 million population)	Annual standardized RTDs (per 1 million population and 1 million vehicles)
2008	391	7.8	1.2	50.1	42.8
2009	532	7.9	1.3	67.2	50.1
2010	671	8.0	1.6	83.4	52.2
2011	705	8.1	1.9	86.7	46.7
2012	731	8.2	2.0	89.1	43.7
2013	674	8.3	2.1	81.2	37.8
2014	677	8.4	2.2	80.6	36.2
2015	553	8.5	2.2	64.7	28.9
2016	544	8.7	2.3	62.5	27.2
2017	575	9.0	2.4	64.0	26.7
2018	483	9.3	2.6	52.1	20.2

*RTDs* road traffic deaths

Table 2 shows that males (74.6%), people aged 16–64 years (72.9%), residents with education of secondary school or above (71.9%) accounted for the majority of RTDs. Blue-collar workers represented 53.2% of the RTDs, while around 70% of the RTDs were pedestrians (33.8%) and motorcyclists (37.5%).

**Table 2:** Summary statistics of road traffic deaths by individual characteristics in Guangzhou, China, 2008–2018

Category	2008–2018		Before May 1, 2011		After May1, 2011	
	Annual number of RTDs^a^	Standardized annual RTDs(per 1 million population and 1 million motor vehicles)	Annual number of RTDs^a^	Standardized annual RTDs (per 1 million population and 1 million motor vehicles)	Annual number of RTDs^a^	Standardized annual RTDs(per 1 million population and 1 million motor vehicles)
All	594.2(100.0)	37.5	546.7(100.0)	48.1	614.8(100.0)	32.9
Sex						
Male	443.2(74.6)	55.3	404.7(74.0)	69.9	459.9(74.8)	49.0
Female	151.0(25.4)	19.3	142.1(26.0)	25.6	154.9(25.2)	16.6
Age, years						
< 16	26.6(4.5)	11.4	28.5(5.2)	16.4	25.8(4.2)	9.2
16–64	432.9(72.9)	37.4	415.5(76.0)	48.9	440.5(71.6)	32.4
≥ 65	134.6(22.7)	75.1	102.7(18.8)	90.0	148.5(24.2)	68.6
Educational attainment^b,c^						
Primary school or below	134.0(22.6)	9.5	200.7(36.7)	17.6	105.1(17.1)	6.0
Secondary school or above	427.5(71.9)	25.6	293.8(53.7)	25.8	485.4(79.0)	25.4
Occupational group^b,c^						
Unemployed	165.9(27.9)	10.7	165.2(30.2)	14.7	166.2(27.0)	9.0
Blue-collar	316.0(53.2)	19.9	291.4(53.3)	25.4	326.7(53.1)	17.5
White-collar	38.5(6.5)	2.5	37.6(6.9)	3.4	39.0(6.3)	2.1
Type of road user^b,c^						
Pedestrian	200.7(33.8)	13.0	209.1(38.2)	18.4	197.1(32.1)	10.7
Motorcyclist	222.9(37.5)	13.7	177.8(32.5)	15.3	242.5(39.4)	12.9
Pedal cyclist	51.0(8.6)	3.1	37.0(6.8)	3.3	57.1(9.3)	3.0
Occupant	64.9(10.9)	4.0	49.3(9.0)	4.3	71.7(11.7)	3.8

*RTDs* road traffic deaths

^a^The numbers in brackets represent the corresponding proportions

^b^We used the total number of residents in Guangzhou to standardize the RTDs for subgroups of different educational attainment, occupational group and type of road user, since the data on population size for these subgroups were not available

^c^The unspecified causes in the educational attainment, occupational group and type of road user were 5.5, 12.4 and 9.2%, respectively

Table 3 presents the estimates of ERs of sRTD associated with drunk driving intervention at three time points: November 19, 2013, June 10, 2016, and December 31, 2018. Compared with the predicted counterfactual, the ERs of sRTD on the three time points were − 38.21% (95% eCI, − 50.07% to − 23.67%), − 59.90% (95% eCI, − 71.44% to − 43.95%) and − 73.98% (95% eCI, − 83.75% to − 58.23%), respectively. We further evaluated the effects of drunk driving intervention on RTDs by individual characteristics. It was found that the ERs of sRTDs were statistically significant for both of males (ER: − 76.18% to − 38.52%) and females (ER: − 66.24% to − 37.42%). The relative risk of sRTDs among residents aged < 16 years and those aged 16–64 years decreased by 62.29 to 93.87% and 39.35 to 76.15% respectively. When stratified by educational attainment, the ERs of sRTDs were statistically significant for education of primary school and below (ER: − 97.94% to − 72.82%). Meanwhile, the effects among the unemployed (ER: − 64.26% to − 26.81%) and blue-collar workers (ER: − 83.55% to − 49.09%) were statistically significant. For different road users, a statistically significant ER was observed in pedestrians (ER: − 82.53% to − 46.91%) and motorcyclists (ER: − 91.00% to − 58.53%).

**Table 3:** Excess risks of road traffic deaths attributable to drunk driving intervention in Guangzhou, China

Category	ER% (95% eCI)		
	November 19, 2013	June 10, 2016	December 31, 2018
All	−38.21(− 50.07 to −23.67)	−59.90(−71.44 to −43.95)	−73.98(− 83.75 to − 58.23)
Sex			
Male	−38.52(−51.63 to −21.68)	− 61.73(−74.01 to −43.24)	−76.18(−86.11 to − 58.56)
Female	−37.42(− 58.80 to − 5.96)	−54.03(−76.49 to −11.36)	−66.24(− 86.73 to −15.42)
Age, years			
< 16	−62.29(−85.18 to − 5.60)	− 84.79(−96.71 to −32.89)	−93.87(−99.27 to − 51.92)
16–64	−39.35(− 52.23 to − 23.31)	−61.97(− 74.08 to −44.57)	− 76.15(− 85.99 to − 59.97)
≥ 65	−26.60(−53.58 to 17.37)	− 40.23(− 71.35 to 25.10)	− 51.33(− 82.28 to 34.99)
Educational attainment			
Primary school or below	−72.82(−81.08 to − 61.02)	−92.52(− 95.83 to − 86.55)	−97.94(− 99.09 to − 95.4)
Secondary school or above	−16.85(− 37.00 to 10.70)	−34.00(−57.63 to 4.17)	−47.62(− 72.01 to − 1.90)
Occupational group			
Unemployed	−26.81(− 49.98 to 7.95)	−48.85(− 71.79 to −5.14)	−64.26(− 84.35 to − 15.73)
Blue-collar	−49.09(− 61.84 to − 31.79)	− 71.07(− 81.73 to − 53.39)	−83.55(− 91.22 to − 68.14)
White-collar	−13.92(− 59.97 to 86.49)	−25.45(− 77.92 to 157.4)	−35.44(− 87.99 to 259.22)
Type of road user			
Pedestrian	−46.91(−61.75 to − 26.66)	− 69.55(− 82.06 to − 48.98)	−82.53(− 91.63 to − 64.30)
Motorcyclist	−58.53(− 71.02 to − 40.37)	−80.68(− 89.11 to − 65.48)	−91.00(− 95.98 to − 79.59)
Pedal cyclist	−24.62(− 65.62 to 65.81)	−42.23(− 83.90 to 103.18)	−55.73(− 92.53 to 148.30)
Occupant	−27.12(− 64.41 to 48.77)	− 50.09(− 84.06 to 55.99)	−65.82(− 92.92 to 66.04)

*eCI* empirical confidence interval; *ER* Excess risk

**Fig. 2:**
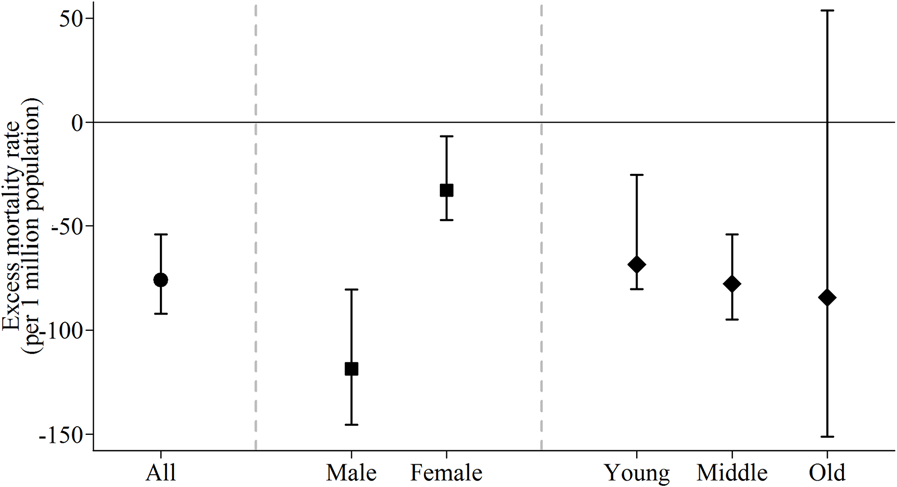
Average annual excess road traffic mortality rate associated with drunk driving intervention in Guangzhou, China. EMR: excess mortality rate. Points and vertical solid lines are point estimates and 95% empirical confidence intervals of EMRs. Young, middle and old represents people aged < 16, 16–64, and ≥ 65 years, respectively

It was estimated that a total of 649.52 RTDs were prevented due to the drunk driving intervention annually, which corresponded to 75.82 (95% eCI, 54.06 to 92.04) per 1 million population (Fig. 2). We estimated that an average of 118.72 (95% eCI, 80.43 to 145.39) RTDs per 1 million males per year were averted due to the introduction of drunk driving regulations since May 2011, greater than in females (EMR: − 32.76, 95% eCI, − 47.16 to − 6.70). The annual EMR was − 68.41 (95% eCI, − 80.18 to − 25.35) and − 77.64 (95% eCI, − 94.86 to − 54.06) for people aged under 16 years and those aged 16–64 years respectively, while the effects for those aged 65 years and older were statistically non-significant (Fig. 2). More details on the baseline trend, level, and slope parameters were provided in Additional file 1.

The result of sensitivity analysis showed that the estimation using the monthly road traffic deaths modeling were roughly the same as the daily data, but with a wider 95% eCI (see Additional file 1). Model I and Model II were used to capture the linear and potential non-linear change in intervention effect over time, respectively. They did not make very much difference, confirming that the change was generally linear (see Additional file 1). Visually inspection of the partial autocorrelation function plots of the residual of the fitted models suggested that the absolute value of partial autocorrelation coefficients lied within the two dashed lines or were less than 0.1 in the first two lag days [19], indicating that the models are appropriate (see Additional file 1).

## Discussion

Our study comprehensively assessed the time-varying effects of drunk driving intervention on road traffic deaths in Guangzhou, China based on interrupted time-series analysis. We estimated that the relative risk of sRTD decreased by 73.98% at the end of the study period due to the enforcement of new drunk driving regulations since May 1, 2011, correspondingly, 650 RTDs (i.e. 76 RTDs per 1 million population) were averted annually. Our analysis presents strong evidence in support of these legislations as a successful public-health intervention that have greatly improved road safety. The estimate of effect of drunk driving laws differs geographically. Our effect estimate of ER of sRTD (− 73.98%) was greater than the estimates for 3.7-year post-intervention period in two other Chinese cities, Ningbo (− 20.0%) [11] and Tianjin (− 11.1%) [12]. The discrepancy in the estimate may be due to the disparities in population density, traffic condition, legal technicality, the degree of regulation enforcement, and the length of post-intervention period investigated.

We observed an apparent continuous decrease in sRTD after the implementation of drunk driving intervention in Guangzhou during the study period. The increasingly strong impacts of drunk driving regulations can be attributable to the introduction of a series of amendments of the drunk driving regulations implemented since May 2011. For instance, the Law on Road Traffic Safety was promulgated in 2011, which detailed the extent of punishment for the drink drivers [20]. The government provided more details on how to define driving after drunkenness, drunk driving and penalties on January 1, 2013 [21]. In addition, the public security bureau and relevant government departments have taken a lot of measures to reduce drunk driving behaviors, including educating the public through TV, radio, newspapers, Internet and other media and platforms, announcing the violations and traffic deaths caused by drunk driving on roadside billboards and electronic screens. Enhanced regulation enforcement after May 2011 further contributes to the increasingly significant impacts of drunk driving regulations. The success of new drunk driving intervention depends not only the detailed descriptions of the definition and the punishment of drunk driving regulations, but also the strict regulation enforcement, such as more frequent and systematic random breath testing of drivers and the cooperation of residents which can be achieved by public education, propaganda, and awareness campaigns [8, 11, 14]. Our findings highlight the importance of integrated intervention measures and strict enforcement for achieving the expected effects.

Previous studies have commonly only used population denominator to standardize road traffic injuries or deaths or even used unstandardized data, and rarely considered the impact of the number of motor vehicles [11, 12, 15, 22, 23]. In Guangzhou, the number of car had an obvious increasing trend during the study period. Therefore, we accounted for both the number of population and motor vehicles as the denominator. In fact, the number of kilometers driven by vehicles would be the ideal denominator for the traffic death rates [15], but these data were generally not available [11, 13]. Haghpanahan et al. [14] used traffic flow as an alternative denominator for the vehicle traveling kilometers. However, multiple imputation with large proportions of missing data in this variable may lead to some bias in the results.

Our findings revealed that the impacts of drunk driving intervention were greater in males (ER = -9.76, 95% eCI: − 10.89% to − 8.29%) than in females (ER = -6.70, 95% eCI: − 8.76% to − 1.75%), which was in accordance with previous studies [12, 22]. This phenomenon might be due to the higher driving and drinking rate among males [24, 25]. As for the age difference, the significant effects of the intervention were observed among people aged < 16 years and 16–64 years, but not among residents aged 65 years and older. Xiong et al. [12] only observed the effect among people aged 16–64 years. The difference in the finding for those aged under 16 years could be due to the higher statistical power from a longer post-intervention period (7.7 years vs. 3.7 years) and the time-varying effects investigated in our study.

It is worth noting that more reductions in RTD associated with drunk driving intervention were observed among those with the education of primary schools or below (ER = -12.60, 95% eCI: − 15.27% to − 10.46%). This might be due to the differential drinking behavior. One study found that the mean consumption of people with the education of primary schools or below was greater than those with higher education (318 g/week vs 241 g/week), and the heaviest drinkers tended to have poorer education [24]. Factor et al. [26] also reported that the probability of involving in severe and fatal accidents increased as drivers’ education level decreased.

Our study also suggested that the impacts of drunk driving intervention differed by occupational groups. It seemed that the intervention effects were higher in blue-collar workers (ER = -9.52, 95% eCI: − 10.68% to − 8.24%) than in the unemployed (ER = -8.99, 95% eCI: − 11.28% to − 4.53%). It is possible that blue-collar workers typically enjoy a better socio-economic status, so they generally choose not to violate traffic laws, which may result in a more favorable response to the intervention [26, 27]. However, There was a low baseline of RTDs and a downward trend before the intervention among white-collar workers. Non-significant intervention effect was found among white-collar workers.

We observed statistically significant effects of drunk driving intervention on road traffic mortality for pedestrians and motorcyclists but not for pedal cyclists or occupants. It could be due to the higher BAC levels and a much higher proportion of alcohol influence among pedestrians and motorcyclists than occupants [28]. In addition, pedestrians and motorcyclists were the most vulnerable to injuries and deaths due to severe head injuries [29, 30].

Our study has three strengths. First, we presented the time-varying effects of the drunk driving intervention on RTDs over a 7.7-year post-intervention period based on daily mortality data. Second, we used the whole population data instead of sampling data or specific subpopulation data to estimate the intervention effects of drunk driving regulations in Guangzhou, avoiding the problems of low coverage rate and representative bias. Third, we considered the differences in intervention effects among different subgroups (e.g. educational attainment, occupational group, and type of road user), which have been seldom examined in previous studies.

There are some limitations of this study. First, we assessed the effectiveness of the new drunk driving intervention only in a single city because of data availability. Further multi-city or national study would provide more substantial evidence to support the promotion of the regulations in other regions. Second, we examined the overall impacts of a series of intervention measures but not a single specific measure alone since it was extremely difficult to distinguish the effects of different parts of an intervention. Third, some subgroups were analyzed in broad categories (e.g., people aged 16–64 years) due to the limited number of RTDs. More data should be collected in the future to increase insights of these subgroups and different subgroup combinations. Finally, some factors, such as road quality and characteristics of the cars possibly have an influence on the number of road traffic deaths. However, these factors were not considered in our analysis since relevant data were not available. More accurate estimate of the intervention effect would be achieved with these data in the future.

## Conclusions

This study conducted quasi-Poisson regression models with an interrupted time-series design to explore the effects of new drunk driving intervention on RTDs. Our finding presents the remarkable time-varying impacts of enforcement of drunk driving intervention since May 2011 in Guangzhou, China. More reductions in RTDs were observed among males, people aged under 16 years and 16–64 years, residents with the education of primary school and below, the unemployed and blue-collar workers, pedestrians, and motorcyclists, compared with their corresponding subgroups. Our findings have important implications for the development of integrated intervention measures for targeted population in China and other countries attempting to reduce traffic fatalities by stricter regulations on drunk driving. In addition, the analytical strategy of our study can be applied to assess the time-varying effect of the intervention on the occurrence of various accidents which change in a linear or non-linear form. The indicators of excess mortality rate would be estimated to better understand the significance of regulations from the public health perspective.

## Supplementary Information

**Additional file 1: Supplement 1.** The R code for the main model. **Table S1.** Regression coefficients and empirical confidence intervals of all RTDs in Guangzhou, China. **Table S2.** Regression coefficients of all RTDs and five subgroups in Guangzhou, China. **Table S3.** Excess risks of monthly road traffic deaths attributable to drunk driving intervention in Guangzhou, China. **Table S4.** Regression coefficients of all monthly RTDs and five subgroups in Guangzhou, China. **Fig. S1.** Excess risks of road traffic deaths over time estimated using the Model II. **Fig. S2.** The partial autocorrelation coefficient plots of the residuals of Model I.

## Data Availability

The data that support the findings of this study are available from Guangzhou Center for Disease Control and Prevention but restrictions apply to the availability of these data, and so the data are not publicly available. We have obtained permission from Guangzhou Center for Disease Control and Prevention to obtain these data. But we have no right to make the original data public. If anyone wants to access the original data, please contact Dr. Pengzhe Qin (email: petgyy@gmail.com) at Guangzhou Center for Disease Control and Prevention. He will instruct you to apply for the permission from Guangzhou CDC.

## Abbreviations

ARIMAX: Autoregressive integrated Moving Average with exogenous variable
BAC: Blood Alcohol Concentration
dfs: degrees of freedom
eCI: empirical Confidence Interval
EMR: Excess Mortality Rate
ER: Excess Risk
ITS: Interrupted Time-Series
RTDs: Road Traffic Deaths
sRTD: Standardized Road Traffic Deaths
