# Supplementary material for: Time-varying effect of drunk driving regulations on road traffic mortality in Guangzhou, China: an interrupted time-series analysis
Source: BMC Public Health. 2021 Oct 19;21:1885. doi: 10.1186/s12889-021-11958-4 (PMC8524860; doi:10.1186/s12889-021-11958-4)

**Supplement 1**: The R code for the main model.

#******************************************************************************

library(timeDate)

library(lubridate)

library(mgcv)

library(splines)

library(tsModel)

library(Epi)

library(dlnm)

library(car)

datafile<-"E:\\RTD\\BMC"

windowsFonts(RMN=windowsFont("Times New Roman"))

RTDdata <- read.csv(file.path(datafile,"RTDdata.csv"),header=T,sep=",")

RTDdata$date<-as.Date(RTDdata$date,origin="1899-12-30")

RTDdata[,43] <-as.numeric(RTDdata[,43])

RTDdata[,44] <-as.numeric(RTDdata[,44])

RTDdata[,45] <-as.numeric(RTDdata[,45])

RTDdata[,46] <-as.numeric(RTDdata[,46])

RTDdata[,47] <-as.numeric(RTDdata[,47])

RTDdata[,48] <-as.numeric(RTDdata[,48])

head(RTDdata)

#------------------------------------------------------------------------------------------------------------------

ER0<-function(model,nsim=5000){

#===ER

coef=as.matrix(ci.lin(model,subset=c("X"),Exp=T)[,1])

cov=vcov(model)[c(2,4),c(2,4)]

I_time<-model.matrix(model)[1217:4018,c(2,4)]

RR=(exp(I_time%*%coef)-1)*100

#===ER95%eCI

coef0<-(c(model$coefficients,1))[c(2,4)]

k0<-length(coef0)

eigen0<-eigen(cov)

X0 <- matrix(rnorm(length(coef0)*nsim),nsim)

coefsim0 <- coef0 + eigen0$vectors %*% diag(sqrt(eigen0$values),k0) %*% t(X0)

ER0<-(exp(I_time%*%coefsim0)-1)*100

ER_elow<-ER_ehigh<-rep(NULL,2802)

for (i in 1:2802){

ER_elow[i] <-sort(ER0[i,])[nsim*0.025]

ER_ehigh[i]<-sort(ER0[i,])[nsim*0.975]

}

result=data.frame(RR,ER_elow,ER_ehigh)

list(result=result)

}

outresult0<-function(model,i,j,k){

modelns <- round(ER0(model)$result,2)

result_e1 <- paste(modelns[i,1],"(",modelns[i,2]," to ",modelns[i,3],")",sep = "")

result_e2 <- paste(modelns[j,1],"(",modelns[j,2]," to ",modelns[j,3],")",sep = "")

result_e3 <- paste(modelns[k,1],"(",modelns[k,2]," to ",modelns[k,3],")",sep = "")

eCI <- data.frame(result_e1,result_e2,result_e3)

list(eCI=eCI)

}

#----------------------------------------------------------------------------------------------------Main model

fourier1 <- harmonic(RTDdata$time,nfreq=1,period=365.25)

cb_temp3 <-crossbasis(RTDdata$temp1,argvar=list(fun="ns",df=3))

RTDdata$Xt<-RTDdata$X*RTDdata$time

model_all0<-glm(RTD~X+time+Xt+Dow+factor(holiday)+fourier1+cb_temp3+offset(log(pop*car)),

data=RTDdata,family=quasipoisson)

summary(model_all0)

res_all0 <- residuals(model_all0,type="deviance")

pacf(res_all0,na.action=na.omit,lag=24)

###

cutoff<-(as.numeric(as.Date("2018-12-31"))-as.numeric(as.Date("2011-05-01")))/3

beginstage <- as.Date(as.numeric(as.Date("2011-05-01"))+cutoff,origin="1970-01-01");beginstage;date1<-as.numeric(beginstage-15094);date1

middlestage <- as.Date(as.numeric(as.Date("2011-05-01"))+cutoff*2,origin="1970-01-01");middlestage;date2<-as.numeric(middlestage-15094);date2

endstage <- as.Date(as.numeric(as.Date("2011-05-01"))+cutoff*3,origin="1970-01-01");endstage;date3<-as.numeric(endstage-15094);date3

as.Date(as.numeric(as.Date("2011-05-01")),origin="1970-01-01");beginstage;middlestage;endstage

#----------------------------------------------------------------------------------------------------Subgroup

subanalys1<-function(response,popcar){

popcar<-popcar

mod1<-eval(parse( text=paste("glm(",response,"~X+time+Xt+Dow+factor(holiday)+cb_temp3+fourier1+offset(log(popcar)),data=RTDdata,family=quasipoisson)",sep="") ))

res1<- residuals(mod1,type="deviance")

result1<-(outresult0(mod1,date1,date2,date3))$eCI

list(mod1=mod1,res1=res1,result1=result1)

}

###

set.seed(20210101)

re_all1<-(outresult0(model_all0,date1,date2,date3))

###

re_male1<-subanalys1("RTD_male",RTDdata$car*RTDdata$popmale)

re_female1<-subanalys1("RTD_fem",RTDdata$car*RTDdata$popfemale)

###

re_151<-subanalys1("RTD_15",RTDdata$car*RTDdata$pop16)

re_16641<-subanalys1("RTD_1664",RTDdata$car*RTDdata$pop1665)

re_651<-subanalys1("RTD_65",RTDdata$car*RTDdata$pop65)

###

re_highed1<-subanalys1("RTD_highed",RTDdata$car*RTDdata$pop)

re_lowed1<-subanalys1("RTD_lowed",RTDdata$car*RTDdata$pop)

###

re_unemp1<-subanalys1("RTD_unemp",RTDdata$car*RTDdata$pop)

re_blue1<-subanalys1("RTD_blue",RTDdata$car*RTDdata$pop)

re_white1<-subanalys1("RTD_white",RTDdata$car*RTDdata$pop)

###

re_pedes1<-subanalys1("RTD_pedes",RTDdata$car*RTDdata$pop)

re_moto1<-subanalys1("RTD_moto",RTDdata$car*RTDdata$pop)

re_cycl1<-subanalys1("RTD_cycl",RTDdata$car*RTDdata$pop)

re_occup1<-subanalys1("RTD_occup",RTDdata$car*RTDdata$pop)

#-----------------------------------------------------------------------------------------------------------Table3

table3<-rbind(

re_all1$eCI,

re_male1$result1 , re_female1$result1,

re_151$result1 , re_16641$result1 , re_651$result1,

re_lowed1$result1 , re_highed1$result1,

re_unemp1$result1 , re_blue1$result1 , re_white1$result1,

re_pedes1$result1 , re_moto1$result1 , re_cycl1$result1 , re_occup1$result1 )

rownames(table3)<-c("All","Male","Female","<16 years","16-64 years",">= 65 years",

"Primary school","Secondary school","Unemployed","Blue-collar","White-collar",

"Pedestrian","Motorcyclist","Pedal cyclist","Occupant")

table3

#------------------------------------------------------------------------------------------------------------PACF

tiff(file =file.path(datafile,"appendfig1.tiff"),height=3500,width=2200,res=300)

par(mfrow=c(5,3),mar=c(5,4,3,1),family="RMN")

pacf(res_all0, na.action=na.omit, lag=25, main="All")

pacf(re_male1$res1, na.action=na.omit, lag=25, main="Male")

pacf(re_female1$res1, na.action=na.omit, lag=25, main="Female")

pacf(re_151$res1, na.action=na.omit, lag=25, main="<16 years")

pacf(re_16641$res1, na.action=na.omit, lag=25, main="16-64 years")

pacf(re_651$res1, na.action=na.omit, lag=25, main=">= 65 years")

pacf(re_lowed1$res1, na.action=na.omit, lag=25, main="Primary school or below")

pacf(re_highed1$res1, na.action=na.omit, lag=25, main="Secondary school or above")

pacf(re_unemp1$res1, na.action=na.omit, lag=25, main="Unemployed")

pacf(re_blue1$res1, na.action=na.omit, lag=25, main="Blue-collar worker")

pacf(re_white1$res1, na.action=na.omit, lag=25, main="White-collar worker")

pacf(re_pedes1$res1, na.action=na.omit, lag=25, main="Pedestrian")

pacf(re_moto1$res1, na.action=na.omit, lag=25, main="Motorcyclist")

pacf(re_cycl1$res1, na.action=na.omit, lag=25, main="Pedal cyclist")

pacf(re_occup1$res1, na.action=na.omit, lag=25, main="Occupant")

dev.off()

#------------------------------------------------------------------------------------------------------------EMR

RTDdata_sub <- RTDdata

fourier1s <- harmonic(RTDdata_sub$time,nfreq=1,period=365.25)

cb_temp3s <-crossbasis(RTDdata_sub$temp1,argvar=list(fun="ns",df=3))

modd<-glm(RTD~X+time+Xt+Dow+factor(holiday)+fourier1s+cb_temp3s+offset(log(pop*car)),data=RTDdata_sub,family=quasipoisson)

data_mod<-as.matrix(data.frame(model.matrix(modd),offset=log(RTDdata$pop*RTDdata$car)))

data_mod0<-data_mod

data_mod0[,c(2,4)]<-0

coef<-c(modd$coefficients,1)

Ddiff<-exp(data_mod %*% coef)-exp(data_mod0 %*% coef)

EMR<-sum(Ddiff[1217:4018])/(245/365+7);EMR

###

EMRanalys<-function(response,nsim,popcar,pop){

popcar<-popcar

mod1<-eval(parse( text=paste("glm(",response,"~X+time+Xt+Dow+factor(holiday)+cb_temp3s+fourier1s+offset(log(popcar)),data=RTDdata_sub,family=quasipoisson)",sep="") ))

#===EMR

data_mod<-as.matrix(data.frame(model.matrix(mod1),offset=log(popcar)))

data_mod0<-data_mod

data_mod0[,c(2,4)]<-0

coef<-c(mod1$coefficients,1)

Ddiff<-exp(data_mod %*% coef)-exp(data_mod0 %*% coef)

EMR<-sum(Ddiff[1217:4018])/(245/365+7)/(sum(unique(pop)[4:11])/8)*1000000

#===EMR95%CI

coef0<-coef[c(2,4)]

k0<-length(coef0)

vcov0<-vcov(mod1)[c(2,4),c(2,4)]

eigen0<-eigen(vcov0)

X0 <- matrix(rnorm(length(coef0)*nsim),nsim)

coefsim0 <- coef0 + eigen0$vectors %*% diag(sqrt(eigen0$values),k0) %*% t(X0)

EMR0<-rep(NULL,nsim)

for(i in 1:nsim){

coef[c(2,4)]<-coefsim0[,i]

dummy<-exp(data_mod %*% coef)-exp(data_mod0 %*% coef)

EMR0[i]<-sum(dummy[1217:4018])/(245/365+7)/(sum(unique(pop)[4:11])/8)*1000000

}

EMRlow <-sort(EMR0)[nsim*0.025]

EMRhigh<-sort(EMR0)[nsim*0.975]

EMRCI<-data.frame(EMR=EMR,EMRlow=EMRlow,EMRhigh=EMRhigh)

list(mod1=mod1,EMRCI=EMRCI)

}

set.seed(20210101)

###

all0<-EMRanalys("RTD",5000,RTDdata_sub$car*RTDdata_sub$pop,RTDdata_sub$pop);all0

###

male1<-EMRanalys("RTD_male",5000,RTDdata_sub$car*RTDdata_sub$popmale,RTDdata_sub$popmale);male1

female1<-EMRanalys("RTD_fem",5000,RTDdata_sub$car*RTDdata_sub$popfemale,RTDdata_sub$popfemale);female1

###

age150<-EMRanalys("RTD_15",5000,RTDdata_sub$car*RTDdata_sub$pop16,RTDdata_sub$pop16);age150

age16640<-EMRanalys("RTD_1664",5000,RTDdata_sub$car*RTDdata_sub$pop1665,RTDdata_sub$pop1665);age16640

age650<-EMRanalys("RTD_65",5000,RTDdata_sub$car*RTDdata_sub$pop65,RTDdata_sub$pop65);age650

#-------------------------------------------------------------------------------------------------------Figure2

EMRdata<-round(rbind(all0$EMRCI,male1$EMRCI,female1$EMRCI,age150$EMRCI,age16640$EMRCI,age650$EMRCI),2)

rownames(EMRdata)<-c("All","Male","Female","Young","Middle","Old")

EMRdata

tiff(file =file.path(datafile,"Line_EMRsub.tiff"),height=1300,width=2200,res=300)

par(mfrow=c(1,1),mar=c(2.5,4,1.0,0.2),mgp=c(1.8,0.4,0),tcl=-0.3,family="RMN")

plot(c(0.3,4.2),c(-70,20),xlab="",xaxt="n",type="n",las=1,ylim=c(-150,50),ylab="",cex.lab=1.2,cex.axis=1.2,bg=1,font.lab=2,family="RMN",bty="L")

abline(v=c(1,2.5),lty=2,lwd=2,col="grey")

axis(side=1,lwd=1.0,at=c(-10,0.5,1.5,2,3,3.5,4),labels=c("",rownames(EMRdata)),cex.axis=1.2,font=1,family="RMN")

axis(2,at=c(-50),labels=c("Excess mortality rate"),pos=-0.17,cex.axis=1.2,tck=0)

axis(2,at=c(-50),labels=c("(per 1 million population)"),cex.axis=1.2,pos=-0.07,tck=0)

a<-c(0.5,1.5,2,3,3.5,4)

for (i in 1:6) {

bg<-c(rep("#D94801",1),rep("#003366",2),rep("#00441B",3))

pch<-c(rep(21,1),rep(22,2),rep(23,3))

points(a[i],EMRdata[i,1],pch=pch[i],bg="black",cex=1.5,lwd=1.2)

lines(c(a[i],a[i]),c(EMRdata[i,2],EMRdata[i,3]),cex=1,lwd=1.8)

lines(c(a[i]-0.03,a[i]+0.03),c(EMRdata[i,2],EMRdata[i,2]),cex=1,lwd=1.8)

lines(c(a[i]-0.03,a[i]+0.03),c(EMRdata[i,3],EMRdata[i,3]),cex=1,lwd=1.8)

}

abline(h=0,lty=1,lwd=1,col="black")

dev.off()

**Table S1**: Regression coefficients and empirical confidence intervals of all RTDs in Guangzhou, China.

| Variable | Coefficient | 95% CI |
| --- | --- | --- |
| (Intercept) | -29.88501 | (-30.12139~-29.64864) * |
| Fourier1 | -0.04663 | (-0.09409~0.00083) |
| Fourier2 | 0.16676 | (0.08000~0.25352) * |
| Temp1 | 0.06906 | (-0.10233~0.24045) |
| Temp2 | 0.37314 | (-0.09606~0.84233) |
| Temp3 | 0.30582 | (0.07725~0.53440) * |
| DowMon | -0.01968 | (-0.11454~0.07518) |
| DowSat | -0.03088 | (-0.12599~0.06424) |
| DowSun | 0.01580 | (-0.07821~0.10980) |
| DowThu | 0.01476 | (-0.07934~0.10886) |
| DowTue | -0.01184 | (-0.10657~0.08290) |
| DowWed | -0.06577 | (-0.16186~0.03031) |
| Holiday1 | -0.06341 | (-0.25421~0.12739) |
| Holiday2 | 0.14176 | (0.03820~0.24532) * |
| X | 0.51385 | (0.36857~0.65913) * |
| t | 0.00014 | (0.00001~0.00028) * |
| Xt | -0.00046 | (-0.00061~-0.00032) * |

**Table S2**: Regression coefficients of all RTDs and five subgroups in Guangzhou, China.

|  | Level change |  | Baseline trend |  | Trend change |  |
| --- | --- | --- | --- | --- | --- | --- |
|  | X  (95% CI) | *P*-value | time  (95% CI) | *P*-value | X:time (95% CI) | *P*-value |
| ALL | 0.51385 (0.36857 to 0.65913) | <0.001 | 0.00014 (0.00001 to 0.00028) | 0.042 | -0.00046 (-0.00061 to -0.00032) | <0.001 |
| Sex |  |  |  |  |  |  |
| Male | 0.60471 (0.43777 to 0.77164) | <0.001 | 0.00017 (0.00001 to 0.00033) | 0.040 | -0.00051 (-0.00067 to -0.00034) | <0.001 |
| Female | 0.24146 (-0.04425 to 0.52718) | 0.098 | 0.00008 (-0.00019 to 0.00036) | 0.540 | -0.00033 (-0.00061 to -0.00005) | 0.021 |
| Age, years |  |  |  |  |  |  |
| <16 | 1.11539 (0.45365 to 1.77713) | <0.001 | 0.00037 (-0.00024 to 0.00099) | 0.232 | -0.00097 (-0.00161 to -0.00033) | 0.003 |
| 16-64 | 0.57442 (0.40676 to 0.74208) | <0.001 | 0.00014 (-0.00002 to 0.00030) | 0.081 | -0.00050 (-0.00066 to -0.00033) | <0.001 |
| ≥65 | 0.16378 (-0.14812 to 0.47567) | 0.303 | 0.00007 (-0.00024 to 0.00038) | 0.652 | -0.00022 (-0.00054 to 0.00010) | 0.173 |
| Educational attainment |  |  |  |  |  |  |
| Primary school or below | 1.66899 (1.37284 to 1.96514) | <0.001 | 0.00025 (0.00001 to 0.00048) | 0.038 | -0.00138 (-0.00164 to -0.00112) | <0.001 |
| Secondary school or above | 0.34746 (0.16260 to 0.53232) | <0.001 | 0.00014 (-0.00004 to 0.00033) | 0.135 | -0.00025 (-0.00044 to -0.00005) | 0.012 |
| Occupational class |  |  |  |  |  |  |
| Unemployed | 0.51285 (0.25067 to 0.77502) | <0.001 | -0.00003 (-0.00027 to 0.00022) | 0.843 | -0.00038 (-0.00064 to -0.00013) | 0.004 |
| Blue-collar | 0.62535 (0.42425 to 0.82644) | <0.001 | 0.00028 (0.00009 to 0.00047) | 0.004 | -0.00060 (-0.00080 to -0.00041) | <0.001 |
| white-collar | 0.18112 (-0.34386 to 0.70610) | 0.499 | -0.00014 (-0.00064 to 0.00036) | 0.584 | -0.00015 (-0.00067 to 0.00037) | 0.562 |
| Type of road user |  |  |  |  |  |  |
| Pedestrian | 0.64633 (0.40918 to 0.88348) | <0.001 | 0.00016 (-0.00006 to 0.00038) | 0.157 | -0.00060 (-0.00082 to -0.00037) | <0.001 |
| Motorcyclist | 0.87755 (0.62464 to 1.13046) | <0.001 | 0.00054 (0.00029 to 0.00079) | <0.001 | -0.00082 (-0.00107 to -0.00056) | <0.001 |
| Pedal cyclist | 0.33019 (-0.18548 to 0.84586) | 0.209 | 0.00016 (-0.00036 to 0.00068) | 0.539 | -0.00029 (-0.00082 to 0.00025) | 0.296 |
| Occupant | 0.55500 (0.08055 to 1.02946) | 0.022 | 0.00018 (-0.00030 to 0.00066) | 0.459 | -0.00041 (-0.00089 to 0.00008) | 0.104 |

RTDs, road trafﬁc deaths; CI, confidence interval.

**Table S3**: Excess risks of monthly road trafﬁc deaths attributable to drunk driving intervention in Guangzhou, China.

| Category | ER% (95% eCI) | | |
| --- | --- | --- | --- |
|  | November 19, 2013 | June 10, 2016 | December 31, 2018 |
| All | -38.96(-52.08 to -22.42) | -60.85(-73.37 to -42.54) | -74.53(-85.09 to -56.56) |
| Sex |  |  |  |
| Male | -39.58(-53.48 to -21.26) | -63.13(-75.87 to -43.01) | -77.13(-87.31 to -58.16) |
| Female | -37.31(-59.92 to -3.02) | -53.49(-77.34 to -5.58) | -65.15(-87.20 to -6.93) |
| Age, years |  |  |  |
| <16 | -61.05(-83.26 to -10.60) | -83.15(-95.80 to -35.03) | -92.51(-98.91 to -51.78) |
| 16-64 | -40.72(-55.19 to -21.89) | -63.73(-76.88 to -43.42) | -77.46(-87.91 to -58.61) |
| ≥65 | -24.91(-52.19 to 19.18) | -37.67(-69.83 to 29.43) | -47.95(-80.86 to 42.01) |
| Educational attainment |  |  |  |
| Primary school or below | -72.42(-83.01 to -55.87) | -92.31(-96.50 to -83.54) | -97.77(-99.26 to -93.56) |
| Secondary school or above | -18.48(-45.53 to 20.85) | -36.77(-66.49 to 19.51) | -50.56(-79.33 to 19.53) |
| Occupational group |  |  |  |
| Unemployed | -28.79(-50.48 to 3.78) | -51.25(-72.65 to -11.37) | -66.22(-84.78 to -23.64) |
| Blue-collar | -49.04(-62.73 to -30.45) | -71.08(-82.46 to -52.41) | -83.29(-91.61 to -67.12) |
| White-collar | -17.37(-62.64 to 85.48) | -30.02(-79.83 to 154.30) | -40.41(-89.19 to 248.42) |
| Type of road user |  |  |  |
| Pedestrian | -46.86(-61.99 to -25.14) | -69.26(-82.13 to -46.13) | -81.90(-91.33 to -60.47) |
| Motorcyclist | -59.46(-72.78 to -39.19) | -81.82(-90.63 to -64.62) | -91.63(-96.67 to -78.91) |
| Pedal cyclist | -25.36(-65.66 to 58.35) | -43.46(-83.76 to 90.22) | -56.79(-92.23 to 132.44) |
| Occupant | -29.90(-65.60 to 43.45) | -53.54(-84.89 to 44.01) | -68.79(-93.43 to 49.98) |

eCI, empirical confidence interval.

**Table S4**: Regression coefficients of all monthly RTDs and five subgroups in Guangzhou, China.

|  | Level change |  | Baseline trend |  | Trend change |  |
| --- | --- | --- | --- | --- | --- | --- |
|  | X  (95% CI) | *P*-value | time  (95% CI) | *P*-value | X:time (95% CI) | *P*-value |
| ALL | 0.52379 (0.35485 to 0.69273) | <0.001 | 0.00464 (-0.00017 to 0.00945) | 0.059 | -0.01433 (-0.01932 to -0.00934) | <0.001 |
| Sex |  |  |  |  |  |  |
| Male | 0.62711 (0.43978 to 0.81443) | <0.001 | 0.00556 (0.00021 to 0.01090) | 0.042 | -0.01593 (-0.02147 to -0.01038) | <0.001 |
| Female | 0.21640 (-0.09767 to 0.53046) | 0.177 | 0.00227 (-0.00661 to 0.01115) | 0.616 | -0.00963 (-0.01884 to -0.00041) | 0.041 |
| Age, years |  |  |  |  |  |  |
| <16 | 0.97664 (0.35570 to 1.59758) | 0.002 | 0.00978 (-0.00714 to 0.02670) | 0.257 | -0.02704 (-0.04475 to -0.00933) | 0.003 |
| 16-64 | 0.60258 (0.40040 to 0.80476) | <0.001 | 0.00487 (-0.00084 to 0.01057) | 0.094 | -0.01585 (-0.02179 to -0.00992) | <0.001 |
| ≥65 | 0.14018 (-0.17377 to 0.45414) | 0.381 | 0.00153 (-0.00772 to 0.01078) | 0.746 | -0.00601 (-0.01553 to 0.00351) | 0.216 |
| Educational attainment |  |  |  |  |  |  |
| Primary school or below | 1.63764 (1.23613 to 2.03914) | <0.001 | 0.00699 (-0.00250 to 0.01648) | 0.149 | -0.04121 (-0.05166 to -0.03075) | <0.001 |
| Secondary school or above | 0.37784 (0.11239 to 0.64329) | 0.005 | 0.00491 (-0.00313 to 0.01294) | 0.231 | -0.00820 (-0.01645 to 0.00005) | 0.051 |
| Occupational class |  |  |  |  |  |  |
| Unemployed | 0.52867 (0.26796 to 0.78939) | <0.001 | -0.00028 (-0.00764 to 0.00708) | 0.940 | -0.01223 (-0.01990 to -0.00456) | 0.002 |
| Blue-collar | 0.62345 (0.40141 to 0.84550) | <0.001 | 0.00843 (0.00216 to 0.01470) | 0.008 | -0.01828 (-0.02479 to -0.01177) | <0.001 |
| white-collar | 0.18978 (-0.36000 to 0.73955) | 0.499 | -0.00359 (-0.01922 to 0.01204) | 0.653 | -0.00536 (-0.02162 to 0.01090) | 0.518 |
| Type of road user |  |  |  |  |  |  |
| Pedestrian | 0.62121 (0.37440 to 0.86802) | <0.001 | 0.00454 (-0.00225 to 0.01133) | 0.190 | -0.01766 (-0.02475 to -0.01056) | <0.001 |
| Motorcyclist | 0.93316 (0.64111 to 1.22521) | <0.001 | 0.01724 (0.00876 to 0.02572) | <0.001 | -0.02586 (-0.03460 to -0.01712) | <0.001 |
| Pedal cyclist | 0.34375 (-0.18020 to 0.86770) | 0.198 | 0.00516 (-0.01063 to 0.02096) | 0.522 | -0.00896 (-0.02518 to 0.00726) | 0.279 |
| Occupant | 0.58683 (0.10307 to 1.07059) | 0.017 | 0.00637 (-0.00804 to 0.02078) | 0.387 | -0.01327 (-0.02811 to 0.00157) | 0.080 |

RTDs, road trafﬁc deaths; CI, confidence interval.

**Figure S1**: Excess risks of road traffic deaths over time estimated using the Model II. ERs: excess risks; RTDs: road traffic deaths. The solid line indicates the point estimates of ERs. The gray shadow represents the 95% empirical confidence intervals of ERs.

**
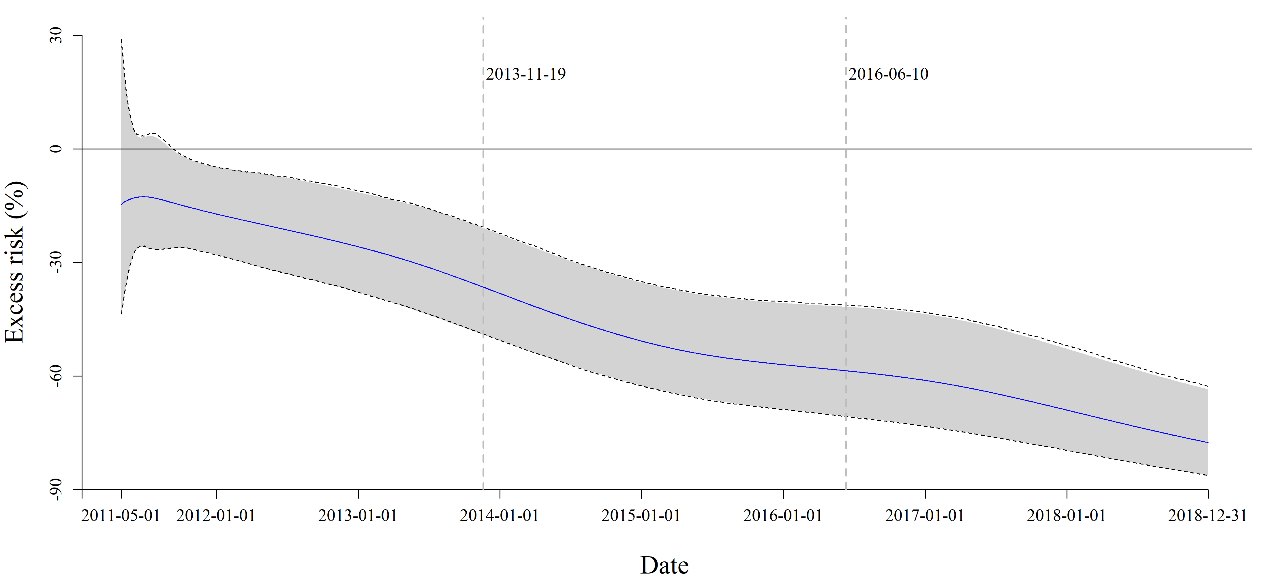
**

**Figure S2**: The partial autocorrelation coefficient plots of the residuals of Model I.


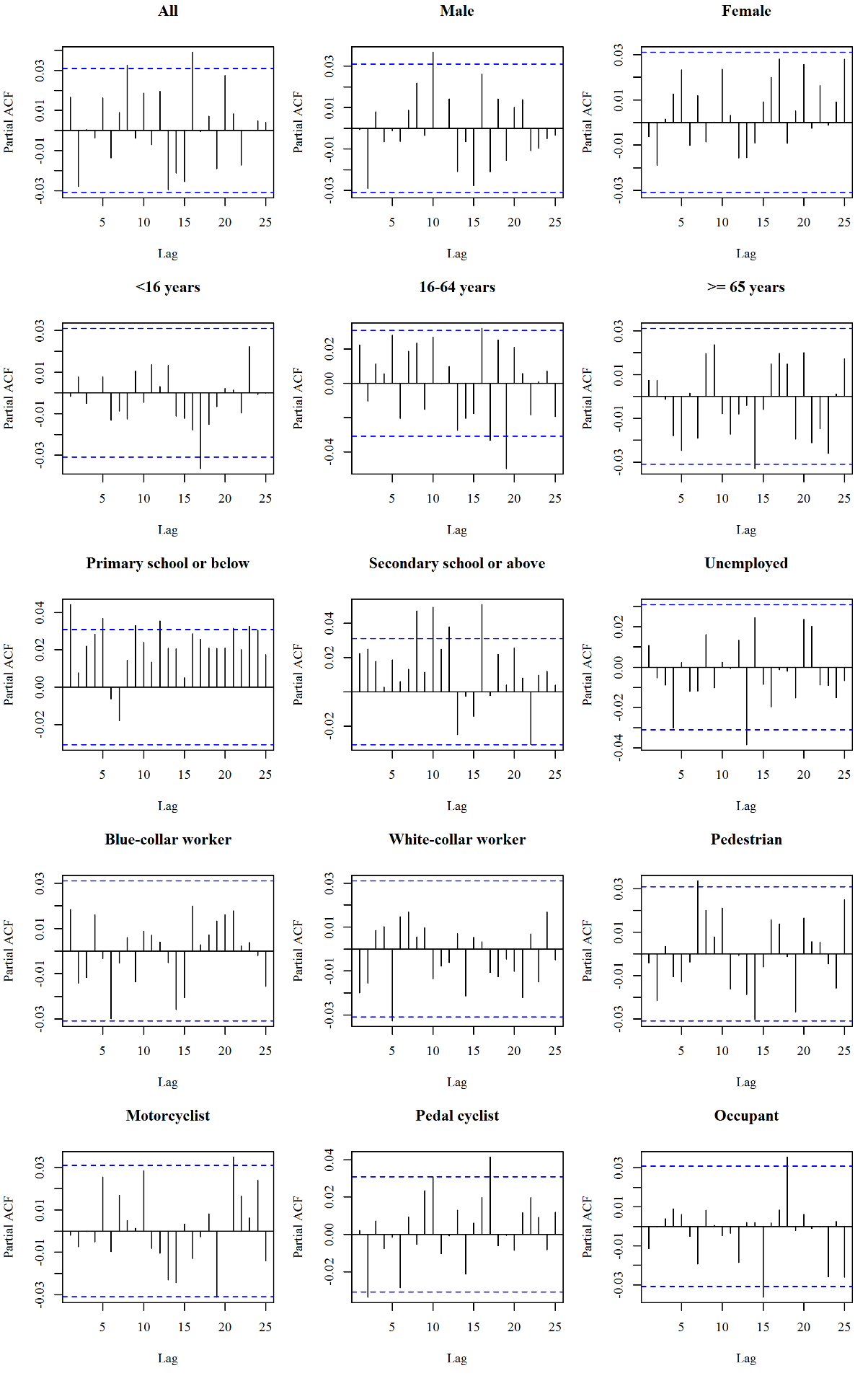

Supplement: Supplementary file 1 — Additional file 1: Supplement 1. The R code for the main model. Table S1. Regression coefficients and empirical confidence intervals of all RTDs in Guangzhou, China. Table S2. Regression coefficients of all RTDs and five subgroups in Guangzhou, China. Table S3. Excess risks of monthly road traffic deaths attributable to drunk driving intervention in Guangzhou, China. Table S4. Regression coefficients of all monthly RTDs and five subgroups in Guangzhou, China. Fig. S1. Excess risks of road traffic deaths over time estimated using the Model II. Fig. S2. The partial autocorrelation coefficient plots of the residuals of Model I. [file 12889_2021_11958_MOESM1_ESM.docx]
